# Difficulties accessing health care services during the COVID-19 pandemic in Canada: examining the intersectionality between immigrant status and visible minority status

**DOI:** 10.1186/s12939-021-01593-1

**Published:** 2021-12-16

**Authors:** Josephine Etowa, Yujiro Sano, Ilene Hyman, Charles Dabone, Ikenna Mbagwu, Bishwajit Ghose, Muna Osman, Hindia Mohamoud

**Affiliations:** 1 Faculty of Health Sciences, University of Ottawa, Ottawa, ON Canada; 2 Department of Sociology, Nipissing University, North Bay, ON Canada; 3 Dalla Lana School of Public Health, University of Toronto, Toronto, ON Canada; 4 Canadians of African Descent Health Organization, Ottawa, ON Canada; 5 Ottawa Local Immigrant Partnership, Ottawa, ON Canada

**Keywords:** COVID-19, Immigrants, Visible minorities, Health care, Canada

## Abstract

**Background:**

Difficulties accessing health care services can result in delaying in seeking and obtaining treatment. Although these difficulties are disproportionately experienced among vulnerable groups, we know very little about how the intersectionality of realities experienced by immigrants and visible minorities can impact their access to health care services since the pandemic.

**Methods:**

Using Statistics Canada’s Crowdsourcing Data: Impacts of COVID-19 on Canadians—Experiences of Discrimination, we combine two variables (i.e., immigrant status and visible minority status) to create a new variable called visible minority immigrant status. This multiplicative approach is commonly used in intersectionality research, which allows us to explore disadvantages experienced by minorities with multiplicative identities.

**Results:**

Main results show that, compared to white native-born, visible minority immigrants are less likely to report difficulties accessing non-emergency surgical care (OR = 0.55, *p* < 0.001), non-emergency diagnostic test (OR = 0.74, *p* < 0.01), dental care (OR = 0.71, *p* < 0.001), mental health care (OR = 0.77, *p* < 0.05), and making an appointment for rehabilitative care (OR = 0.56, *p* < 0.001) but more likely to report difficulties accessing emergency services/urgent care (OR = 1.46, *p* < 0.05).

**Conclusion:**

We conclude that there is a dynamic interplay of factors operating at multiple levels to shape the impact of COVID-19 related needs to be addressed through changes in social policies, which can tackle unique struggles faced by visible minority immigrants.

## Introduction

Since the beginning of the COVID-19 pandemic, health care system resources are disproportionately demanded by COVID-19 patients in Canada, often creating an environment where it is difficult to meet the health care needs of non-COVID-19 patients [1]. This is unfortunate, considering that easy and timely access to health care services is essential for maintaining health and well-being. Using the 2013 Canadian Community Health Survey, Clarke [2] reveals that about 30% of Canadians with health care needs reported difficulties accessing health care services such as ‘waiting too long for an appointment’ and ‘difficulty getting an appointment’. This finding is concerning because Canadians with these experiences may delay seeking and obtaining treatment, underuse primary health care services, and be exposed to a greater risk for the complications of delayed diagnoses [3]. Despite the emphasis on health equity of the Health Canada Act, research points to a disproportionate burden of difficulties in accessing health care services among vulnerable populations in Canada, such as women, rural residents, Indigenous people, African, Caribbean, and Black (ACB) people and people with health problems [2–6].

Research also shows that immigrant status is a critical factor in difficulties accessing health care services. For example, compared to the native-born, recent immigrants (who have been in Canada for less than 5 years) are more likely to have difficulties accessing immediate care but established immigrants (5 years or more in Canada) are less likely to face difficulties accessing routine care [2]. Harrington et al. [6] also point out that both recent and established immigrants are more likely to experience difficulties accessing specialist care than the native-born. Moreover, the odds of difficulties accessing specialized services are higher for immigrants than the native-born; however, a significant difference is not observed for immediate care [2]. Immigrants also report higher rates of difficulties accessing infant health care than the native-born [7]. These findings are worrying, particularly in the context of the ‘healthy immigrant effect’, whereby immigrants are healthier than the native-born at the time of their arrival in Canada; however, their health advantage often disappears within 10 years [8].

Although these studies are useful, the role of visible minority status is largely missing in the literature on difficulties accessing health care services. According to the Employment Equity Act, visible minorities are defined as ‘persons other than Aboriginal peoples who are non-Caucasian in race or non-White in colour’, including Chinese, South Asians, Blacks, Arabs/West Asians, Filipinos, Japanese, Koreans, and others. Visible minorities account for 22% of the total population and mostly include immigrants, their children, and grandchildren [9]. Visible minorities often have more unique cultural, linguistic, social, and economic characteristics than their non-visible minority counterparts. For example, Hou et al. [10] find that the COVID-19 pandemic serves as a greater barrier to meeting financial obligations or essential needs for visible minorities than whites. Similarly, Chinese, Korean, Southeast Asian, and Black people are twice or more likely to report perceived discrimination than whites since the beginning of the pandemic [11]. Thus, it is important to extend our analysis to reflect these visible minorities’ experiences of difficulties accessing health care services.

In the current study, we aim to incorporate both immigrant status and visible minority status in the analysis of difficulties accessing health care services. While it is common for quantitative research to assess the independent impact of immigrant status and visible minority status on health and health care utilization, the literature suggests that this approach overlooks the complexity of the intersectionality between immigrant status and visible minority status [12, 13]. The importance of this approach can be further highlighted by the theory of intersectionality, which seeks to examine how various socially and culturally constructed categories (such as immigrant status, visible minority status, sexual orientation, and religious identity) do not act independently but rather interact on multiple levels, creating a system of oppression that contributes to inequalities in society [14].

Accordingly, it is possible that the intersectionality of realities experienced by immigrants and visible minorities can impact their access to health care services since the pandemic. Specifically, navigating through a new health care system can be difficult for all immigrants, particularly for visible minority immigrants, as many of them migrate from less-developed regions where health care systems are different than the one in Canada [15]. Overcoming this barrier may be persistently challenging for visible minority immigrants when they concomitantly face other barriers such as language problems, racial discrimination, and lack of cultural competence in health care [16]. As health care systems have been exposed to increasing demand for care of COVID-19 patients, visible minority immigrants’ difficulties accessing health care services may be more intensified by these experiences. To this end, we compare difficulties accessing ten types of health care services among non-visible minority native-born, visible minority native-born, non-visible minority immigrants, and visible minority immigrants.

## Method

We draw on data from Statistics Canada’s Crowdsourcing Data: Impacts of COVID-19 on Canadians—Experiences of Discrimination. Respondents were recruited online through open advertisement between August 4 to 24, 2020, with self-selection as a sampling methodology. The survey covers two main topics: 1) experiences of discrimination based on race, sex, gender identity or expression, ethnicity, religion, sexual orientation, age, disability, and language and 2) impacts of COVID-19 on experiences of discrimination and trust in various institutions, the public, and neighbours. The target population includes all Canadians aged 15 and up living in one of the ten provinces or three territories during the collection period. Crowdsourcing data did not use a probabilistic approach to collecting data. Therefore, unlike other surveys conducted by Statistics Canada, a survey weight cannot be calculated. Instead, benchmarking techniques are applied to correct for unbalanced responses across sociodemographic characteristics. More information about this survey can be found in the Statistics Canada report [11].

### Dependent variables

The survey asked respondents if they experienced difficulties accessing the following services since the beginning of the COVID-19 pandemic: 1) non-emergency surgery, 2) non-emergency diagnostic test, 3) appointment with a family doctor, 4) appointment with a medical specialist, 5) appointment for rehabilitative care, 6) dental care, 7) mental health care, 8) medical treatment, 9) natural medicine, and 10) emergency services/urgent care. We use these ten variables as the dependent variables to capture a comprehensive understanding of difficulties accessing health care services. There are three potential response categories for each service: a) yes, b) no, and c) I do not need the service. To identify difficulties accessing health care services, we exclude respondents who did not need the service and code ‘yes’ as a higher category in the variable (0 = no; 1 = yes).

### Independent variable

Guided by the theory of intersectionality, the independent variable captures the combination of realities experienced by immigrants and visible minorities. In this case, a traditional additive approach, which assumes privileges and oppressions experienced by different groups can be separated and treated independently, may not be suitable [14]. Thus, instead of adding immigrant status and visible minority status separately as two independent variables, as shown in Table 1, we combine immigrant status (0 = native-born; 1 = immigrant) and visible minority status (0 = non-visible minority; 1 = visible minority) and generate a new variable called ‘visible minority immigrant status’ (0 = non-visible minority native-born; 1 = visible minority native-born; 2 = non-visible minority immigrant; 3 = visible minority immigrant). This multiplicative approach is commonly used in intersectionality research, which allows us to explore unique social locations held by some minorities with multiplicative identities [17].

**Table 1:** Categorization of visible minority immigrant status

		Immigrant status	
		Native-born	Immigrant
**Visible minority status**	**Non-visible minority**	Native-born non-visible minority	Non-visible minority immigrant
	**Visible minority**	Visible minority native-born	Visible minority immigrant

### Control variables

To account for potential confounding factors, we introduce factors informed by the Andersen and Newman model of health service use [18]. According to this framework, people’s use of health care services is impacted by three different blocks of factors, including predisposing, enabling, and need factors. Predisposing factors capture social structure, health system beliefs, and demographic characteristics. Accordingly, we include experience of discrimination (0 = no; 1 = yes), trust towards health care system (0 = great deal of trust; 1 = fair amount of trust; 2 = moderate amount of trust; 3 = little trust; 4 = no trust at all), education (0 = no university; 1 = university), sex (0 = male; 1 = female), rural residence (0 = no; 1 = yes), age (0 = 15–34; 1 = 35–44; 2 = 45+; 3 = unknown), marital status (0 = married; 1 = not married), LGBTQ2 (0 = no; 1 = yes), Indigenous identity (0 = no; 1 = yes), and living arrangement (0 = living alone; 1 = multiple person household without children; 2 = multiple person household with children). Enabling factors reflect economic and social resources that enable people to use health care services. Unfortunately, the survey does not have an indicator of income that is often included as part of economic resources. We include a sense of belonging to a community (0 = very strong; 1 = somewhat strong; 2 = somewhat weak; 3 = very weak) as a type of social resources. Finally, the most immediate cause of health service use may contain functional and health problems that lead to the need for health care utilization. To account for this need factor, we add any difficulty or long-term condition (i.e., difficulty seeing, even when wearing glasses/contact lenses; difficulty hearing, even when using hearing aid/cochlear implant; difficulty walking, using stairs, using hands or fingers/other physical act; difficulty learning, remembering, concentrating; emotional, psychological, mental health conditions; or other health problem/long-term condition) that has lasted or are expected to last for six or more months (0 = no; 1 = yes).

### Statistical analysis

Although originally collected from 36,674 respondents, as we exclude the respondents who did not need the health care service from the analysis, the sample size differs for each type of health care. The sample size for each type of health care is shown in Table 3. We do not present the characteristics of analytical sample for each heath care service in this paper; however, these estimates are available based on request. Consequently, we show the characteristics of the respondents who needed at least one type of health care service (see Table 2). We then describe the bivariate results between the dependent and independent variables, describing relevant percentages and odds ratios (see Table 3). We also employ multivariate analysis for the outcomes significantly associated with the independent variable at the bivariate level (i.e., non-emergency surgery, non-emergency diagnostic test, appointment for rehabilitative care, dental care, mental health care, and emergency services/urgent care) by accounting for theoretically relevant variables (i.e., predisposing, enabling, and need factors) to estimate odds ratios, describing the net impact of the independent variable on the dependent variable (see Table 4). Finally, as a subsequent analysis, we explore whether the reasons for difficulties accessing health care services differ based on visible minority immigrant status with relevant percentages and odds ratios (see Table 5). In particular, we examine whether we observe significant disparities with the reasons for difficulties accessing health care services such as 1) getting a referral, 2) getting an appointment, 3) contacting physicians/nurses for information, 4) waiting between booking and visit, 5) waited too long to get service, 6) not available at time required, 7) refused for symptoms of COVID-19, 8) transportation problems, 9) language problems, 10) cost, and 11) other reasons. Consistent with previous research [6, 19], we rely on bivariate statistics for this part of analysis. As all dependent variables are binary in the nature, we use logistic regression analysis for multivariate analysis. In all analyses, *p* < 0.05 is set as a cut-off for statistical significance. All analyses are conducted using Stata 13.1 (StataCorp, College Station, TX).

**Table 2:** Characteristics of the respondents who needed at least one type of health care service

	Percentage
**Health care services accessibility**	
No issues in any service	38 (37, 38)
Issues with one service	22 (22, 23)
Issues with multiple services	40 (39, 41)
**Visible minority immigrant status**	
Non-visible minority native-born	71 (70, 71)
Visible minority native-born	8 (8, 9)
Non-visible minority immigrants	7 (7, 8)
Visible minority immigrants	14 (13, 14)
**Experience of discrimination**	
No	70 (70, 71)
Yes	30 (29, 30)
**Trust towards health care system**	
Great deal of trust	24 (23, 24)
Fair amount of trust	40 (39, 41)
Moderate amount trust	23 (22, 24)
Little amount of trust	10 (9, 10)
No trust at all	3 (3, 4)
**Level of education**	
No university	36 (35, 36)
University	64 (64, 65)
**Sex**	
Male	47 (47, 48)
Female	53 (52, 53)
**Rural residence**	
No	91 (91, 92)
Yes	9 (8, 9)
**Age of respondents**	
15–34	25 (24, 26)
35–44	13 (13, 14)
45+	54 (53, 55)
Unknown	8 (7, 8)
**Marital status**	
Married	63 (62, 64)
Not married	37 (36, 38)
**LGBTQ2**	
No	86 (85, 86)
Yes	14 (14, 15)
**Indigenous identity**	
No	97 (97, 97)
Yes	3 (3, 3)
**Living arrangement**	
Living alone	17 (17, 18)
Multiple persons, no children	54 (53, 54)
Multiple persons, with children	29 (29, 30)
**Sense of belonging to the community**	
Very strong	28 (27, 29)
Somewhat strong	41 (40, 41)
Somewhat weak	24 (23, 24)
Very weak	8 (7, 8)
**Any health problem/long-term conditions**	
No	49 (48, 50)
Yes	51 (50, 52)
Total	28,800

Point estimates and 95% confidence intervals (in parentheses)

**Table 3:** Difficulties accessing each type of health care service by visible minority immigrant status

	Overall	NVMNB	VMNB	NVMI	VMI
**Non-emergency surgery (*n*** **= 5352)**					
Percentage	35 (34, 37)	37 (36, 39)	37 (30, 45)	35 (30, 42)	24 (20, 29)
Odds ratio		1.00	0.97 (0.70, 1.37)	0.92 (0.69, 1.21)	0.54 (0.41, 0.70)***
**Non-emergency diagnostic test (*n*** **= 11,046)**					
Percentage	39 (38, 40)	40 (39, 41)	37 (32, 43)	39 (35, 44)	36 (32, 39)
Odds ratio		1.00	0.88 (0.69, 1.12)	0.97 (0.81, 1.17)	0.83 (0.70, 0.98)*
**Appointment with family doctor (*n*** **= 21,954)**					
Percentage	37 (36, 38)	37 (36, 38)	38 (34, 41)	35 (32, 38)	38 (35, 41)
Odds ratio		1.00	1.04 (0.88, 1.24)	0.95 (0.82, 1.09)	1.06 (0.94, 1.20)
**Appointment with medical specialist (*n*** **= 13,875)**					
Percentage	46 (45, 47)	46 (45, 48)	45 (40, 50)	43 (39, 47)	44 (41, 48)
Odds ratio		1.00	0.96 (0.78, 1.18)	0.88 (0.75, 1.04)	0.91 (0.79, 1.06)
**Appointment for rehabilitative care (*n*** **= 10,127)**					
Percentage	44 (43, 45)	45 (44, 47)	48 (42, 54)	46 (41, 51)	33 (30, 37)
Odds ratio		1.00	1.11 (0.87, 1.41)	1.04 (0.84, 1.28)	0.60 (0.50, 0.73)***
**Dental care (*n*** **= 20,055)**					
Percentage	48 (47, 49)	50 (48, 51)	45 (41, 49)	46 (43, 50)	44 (41, 47)
Odds ratio		1.00	0.82 (0.69, 0.97)*	0.88 (0.77, 1.01)	0.79 (0.69, 0.89)***
**Mental health care (*n*** **= 9681)**					
Percentage	41 (39, 42)	42 (40, 43)	45 (40, 51)	37 (32, 42)	35 (31, 39)
Odds ratio		1.00	1.16 (0.92, 1.45)	0.83 (0.65, 1.04)	0.76 (0.62, 0.92)**
**Medical treatment (*n*** **= 4438)**					
Percentage	20 (18, 21)	20 (19, 22)	20 (14, 27)	20 (15, 26)	18 (15, 23)
Odds ratio		1.00	0.97 (0.62, 1.50)	0.97 (0.68, 1.40)	0.89 (0.66, 1.20)
**Natural medicine (*n*** **= 4820)**					
Percentage	23 (22, 25)	24 (22, 25)	25 (19, 32)	26 (21, 33)	21 (17, 26)
Odds ratio		1.00	1.08 (0.76, 1.55)	1.15 (0.83, 1.59)	0.87 (0.66, 1.15)
**Emergency services/urgent care (*n*** **= 6369)**					
Percentage	19 (18, 20)	18 (17, 20)	21 (15, 28)	14 (10, 19)	24 (20, 28)
Odds ratio		1.00	1.18 (0.80, 1.75)	0.73 (0.51, 1.04)	1.42 (1.11, 1.82)**

*p < 0.05, **p < 0.01, ***p < 0.001; *NVMNB* non-visible minority native-born, *VMNB* visible minority native-born, *NVMI* non-visible minority immigrants, *VMI* visible minority immigrants; odds ratio obtained from logistic regression; point estimates and 95% confidence intervals (in parentheses)

**Table 4:** Multivariate analysis of difficulties accessing health care services

	NES	NEDT	ARC	DC	MHC	ESUC
	OR (95% CI)	OR (95% CI)	OR (95% CI)	OR (95% CI)	OR (95% CI)	OR (95% CI)
**Visible minority immigrant status**						
NVMNB	1.00	1.00	1.00	1.00	1.00	1.00
VMNB	0.96 (0.66, 1.40)	0.74 (0.57, 0.96)*	0.99 (0.77, 1.28)	0.69 (0.58, 0.83)***	0.98 (0.75, 1.27)	1.12 (0.74, 1.69)
NVMI	0.99 (0.74, 1.32)	1.02 (0.84, 1.24)	1.05 (0.85, 1.30)	0.92 (0.80, 1.06)	1.03 (0.80, 1.32)	0.87 (0.60, 1.25)
VMI	0.55 (0.40, 0.75)***	0.74 (0.61, 0.89)***	0.56 (0.46, 0.68)***	0.71 (0.62, 0.82)***	0.77 (0.62, 0.96)*	1.46 (1.07, 1.98)*
**Experience of discrimination**						
No	1.00	1.00	1.00	1.00	1.00	1.00
Yes	1.42 (1.18, 1.71)***	1.50 (1.33, 1.70)***	1.39 (1.22, 1.58)***	1.41 (1.28, 1.56)***	1.87 (1.63, 2.15)***	1.72 (1.41, 2.09)***
**Trust towards health care**						
Great deal of trust	1.00	1.00	1.00	1.00	1.00	1.00
Fair amount of trust	1.12 (0.89, 1.41)	1.36 (1.17, 1.58)***	1.15 (0.98, 1.35)	1.06 (0.95, 1.18)	1.01 (0.83, 1.23)	1.20 (0.89, 1.63)
Moderate amount trust	1.39 (1.08, 1.80)*	1.76 (1.49, 2.07)***	1.21 (1.02, 1.45)*	1.23 (1.09, 1.38)**	1.15 (0.94, 1.42)	2.29 (1.68, 3.10)***
Little amount of trust	1.95 (1.45, 2.63)***	2.93 (2.38, 3.60)***	1.67 (1.34, 2.07)***	1.50 (1.29, 1.76)***	1.90 (1.50, 2.42)***	3.76 (2.67, 5.29)***
No trust at all	2.73 (1.83, 4.08)***	4.86 (3.61, 6.54)***	1.72 (1.26, 2.34)***	1.64 (1.28, 2.10)***	2.33 (1.69, 3.23)***	7.07 (4.76, 9.51)***
**Level of education**						
No university	1.00	1.00	1.00	1.00	1.00	1.00
University	1.01 (0.85, 1.20)	1.18 (1.05, 1.33)**	1.13 (0.99, 1.28)	1.12 (1.02, 1.22)*	0.85 (0.74, 0.98)*	1.05 (0.86, 1.28)
**Sex**						
Male	1.00	1.00	1.00	1.00	1.00	1.00
Female	1.18 (0.99, 1.39)	1.27 (1.13, 1.43)***	1.45 (1.28, 1.65)***	1.08 (0.99, 1.17)	1.25 (1.08, 1.46)**	1.16 (0.94, 1.42)
**Rural residence**						
No	1.00	1.00	1.00	1.00	1.00	1.00
Yes	1.13 (0.88, 1.44)	1.11 (0.92, 1.32)	1.25 (1.03, 1.52)**	1.23 (1.08, 1.41)**	1.30 (1.04, 1.61)*	1.04 (0.78, 1.38)
**Age of respondents**						
15–34	1.00	1.00	1.00	1.00	1.00	1.00
35–44	0.98 (0.75, 1.27)	1.02 (0.85, 1.23)	1.40 (1.18, 1.67)***	0.98 (0.86, 1.11)	0.73 (0.61, 0.87)***	0.85 (0.64, 1.12)
45+	1.18 (0.94, 1.50)	1.05 (0.89, 1.23)	1.35 (1.15, 1.58)***	0.95 (0.84, 1.06)	0.74 (0.64, 0.87)***	1.03 (0.80, 1.33)
Unknown	1.06 (0.66, 1.70)	1.03 (0.75, 1.40)	1.86 (1.35, 2.56)***	1.13 (0.92, 1.39)	1.24 (0.91, 1.68)	1.24 (0.79, 1.94)
**Marital status**						
Married	1.00	1.00	1.00	1.00	1.00	1.00
Not married	0.94 (0.75, 1.16)	0.96 (0.82, 1.12)	0.96 (0.81, 1.13)	0.97 (0.86, 1.09)	1.18 (1.01, 1.39)*	0.98 (0.77, 1.24)
**LGBTQ2**						
No	1.00	1.00	1.00	1.00	1.00	1.00
Yes	0.93 (0.72, 1.21)	1.09 (0.91, 1.31)	1.11 (0.92, 1.33)	1.17 (1.02, 1.33)*	1.33 (1.12, 1.57)**	1.10 (0.83, 1.45)
**Indigenous identity**						
No	1.00	1.00	1.00	1.00	1.00	1.00
Yes	1.05 (0.73, 1.51)	1.02 (0.77, 1.35)	0.92 (0.67, 1.26)	0.96 (0.77, 1.19)	1.12 (0.86, 1.47)	1.81 (1.26, 2.59)**
**Living arrangement**						
Living alone	1.00	1.00	1.00	1.00	1.00	1.00
Multiple persons, no children	1.02 (0.77, 1.34)	1.21 (0.99, 1.48)	1.00 (0.82, 1.22)	1.19 (1.03, 1.37)*	1.13 (0.92, 1.38)	1.08 (0.78, 1.49)
Multiple persons, with children	1.15 (0.86, 1.53)	1.17 (0.95, 1.44)	1.17 (0.94, 1.44)	1.20 (1.03, 1.39)*	1.49 (1.20, 1.84)***	1.33 (0.96, 1.86)
**Sense of belonging**						
Very strong	1.00	1.00	1.00	1.00	1.00	1.00
Somewhat strong	1.43 (1.19, 1.73)***	1.18 (1.03, 1.34)*	1.24 (1.09, 1.43)**	1.11 (1.01, 1.22)*	1.12 (0.95, 1.33)	1.06 (0.84, 1.33)
Somewhat weak	1.45 (1.15, 1.83)**	1.31 (1.12, 1.53)***	1.57 (1.32, 1.85)***	1.27 (1.13, 1.43)***	1.40 (1.16, 1.69)***	1.14 (0.88, 1.49)
Very weak	1.64 (1.20, 2.25)**	1.46 (1.16, 1.84)***	1.43 (1.13, 1.80)**	1.33 (1.12, 1.58)**	1.76 (1.39, 2.23)***	1.19 (0.85, 1.67)
**Any health problem**						
No	1.00	1.00	1.00	1.00	1.00	1.00
Yes	3.23 (2.70, 3.86)***	2.21 (1.96, 2.48)***	2.18 (1.94, 2.46)***	1.54 (1.42, 1.67)***	2.85 (2.42, 3.35)***	2.03 (1.64, 2.50)***
Wald X2	371.83***	652.66***	437.49***	400.77***	598.60***	391.62***

*NES* non-emergency surgery, *NEDT* non-emergency diagnostic test, *DC* dental care, *MHC* mental health care, *ESUC* emergency service/urgent care; *p < 0.05, **p < 0.01, ***p < 0.001; point estimates and 95% confidence intervals (in parentheses)

**Table 5:** Reasons for difficulties accessing health care services by visible minority immigrant status

	Percentage				
	Overall	NVMNB	VMNB	NVMI	VMI
**Getting a referral**					
Percentage	18 (18, 19)	18 (17, 19)	21 (18, 25)	18 (15, 21)	21 (18, 24)
Odds ratio		1.00	1.24 (0.98, 1.57)	0.99 (0.81, 1.23)	1.22 (1.02, 1.46)*
**Getting an appointment**					
Percentage	69 (68, 70)	69 (68, 70)	67 (63, 72)	67 (64, 71)	68 (65, 71)
Odds ratio		1.00	0.92 (0.75, 1.13)	0.91 (0.77, 1.08)	0.93 (0.80, 1.09)
**Contacting physicians/nurses for information**					
Percentage	29 (28, 29)	27 (26, 28)	31 (27, 36)	29 (25, 32)	33 (30, 37)
Odds ratio		1.00	1.21 (0.99, 1.48)	1.07 (0.90, 1.28)	1.33 (1.15, 1.55)***
**Waiting between booking and visit**					
Percentage	32 (31, 33)	30 (29, 31)	34 (30, 39)	31 (27, 34)	40 (37, 43)
Odds ratio		1.00	1.20 (0.98, 1.48)	1.03 (0.87, 1.22)	1.55 (1.34, 1.80)***
**Waited too long to get service**					
Percentage	16 (15, 16)	14 (14, 15)	17 (14, 21)	13 (11, 16)	22 (19, 25)
Odds ratio		1.00	1.21 (0.93, 1.56)	0.90 (0.72, 1.14)	1.69 (1.41, 2.03)***
**Not available at time required**					
Percentage	46 (45, 47)	47 (46, 48)	49 (44, 53)	41 (38, 45)	43 (40, 47)
Odds ratio		1.00	1.06 (0.88, 1.29)	0.79 (0.67, 0.92)**	0.85 (0.74, 0.98)*
**Refused for symptoms of COVID-19**					
Percentage	3 (2, 3)	3 (2, 3)	3 (2, 7)	2 (1, 3)	3 (2, 4)
Odds ratio		1.00	1.58 (0.89, 2.80)	0.63 (0.38, 1.06)	1.06 (0.68, 1.67)
**Transportation problems**					
Percentage	7 (6, 8)	6 (6, 7)	8 (6, 10)	5 (4, 7)	11 (9, 14)
Odds ratio		1.00	1.24 (0.89, 1.72)	0.83 (0.59, 1.15)	1.81 (1.36, 2.40)***
**Language problems**					
Percentage	1 (1, 2)	1 (1, 2)	1 (1, 3)	1 (1, 2)	3 (2, 5)
Odds ratio		1.00	1.01 (0.41, 2.50)	0.66 (0.31, 1.43)	2.68 (1.67, 4.30)***
**Cost**					
Percentage	10 (9, 10)	9 (8, 10)	12 (10, 16)	6 (5, 9)	13 (10, 15)
Odds ratio		1.00	1.44 (1.07, 1.95)*	0.68 (0.50, 0.93)*	1.43 (1.13, 1.81)**
**Other**					
Percentage	16 (15, 17)	16 (15, 17)	16 (13, 20)	15 (13, 18)	14 (12, 16)
Odds ratio		1.00	0.98 (0.75, 1.28)	0.92 (0.74, 1.15)	0.83 (0.68, 1.02)
**Total**	16,302	13,027	825	1160	1290

*p < 0.05, **p < 0.01, ***p < 0.001; *NVMNB* non-visible minority native-born, *VMNB* visible minority native-born, *NVMI* non-visible minority immigrants, *VMI* visible minority immigrants

## Results

Table 2 describes the characteristics of the respondents who needed at least one type of health care service. It is interesting that more than three fifths of respondents (62%) report that they face difficulties in accessing at least one type of health care service. We also find the largest visible minority immigrant group is non-visible native-born (71%), followed by visible minority immigrants (14%), visible minority native-born (8%), and non-visible immigrants (7%). Approximately one-third of respondents (30%) report that they have experienced discrimination. In addition, the majority of respondents are university-educated (64%) and married (63%). Importantly, approximately half of the respondents (51%) report that they have at least one health problem or long-term condition.

Table 3 shows the findings from bivariate analysis that examines whether the difficulties accessing health care services differ based on visible minority immigrant status. We find that compared to non-visible native-born (37%), visible minority immigrants (24%) are less likely to report difficulties accessing non-emergency surgery (OR = 0.54, *p* < 0.001). Similarly, compared to non-visible minority native-born (40%), visible minority immigrants (36%) are less likely to report difficulties accessing non-emergency diagnostic test (OR = 0.83, *p* < 0.05). Also, the percentage of visible minority immigrants (33%) reporting difficulties making an appointment for rehabilitative care is also smaller than that of non-visible minority native-born (45%) (OR = 0.60, *p* < 0.001). In terms of dental care, compared to non-visible minority native-born (50%), visible minority immigrants (44%) are also less likely to report difficulties accessing services (OR = 0.79, *p* < 0.001). We observe a similar trend for mental health care, indicating that, compared to non-visible minority native-born (42%), visible minority immigrants (35%) are less likely to report difficulties accessing services. In contrary to these findings, visible minority immigrants (24%) are more likely to report difficulties accessing emergency services/urgent care than non-visible minority native-born (18%) (OR = 1.42, *p* < 0.01).

For the significant bivariate associations observed in Table 3, we further account for predisposing, enabling, and need factors. Table 4 shows these multivariate results, which are largely consistent with bivariate results. Specifically, compared to non-visible native-born, visible minority immigrants are less likely to report difficulties accessing non-emergency surgery (OR = 0.55, *p* < 0.001), non-emergency diagnostic test (OR = 0.74, *p* < 0.001), dental care (OR = 0.71, *p* < 0.001), and mental health care (OR = 0.77, *p* < 0.05). Visible minority immigrants are less likely to face difficulties making an appointment for rehabilitative care than non-visible minority native-born (OR = 0.56, *p* < 0.001). Visible minority native-born are also less likely to report difficulties accessing dental care than their non-visible minority counterparts (OR = 0.69, *p* < 0.001). In addition, we find that visible minority immigrants are still more likely to report difficulties accessing emergency services/urgent care than non-visible minority native-born; however, this difference is partially explained by theoretically relevant variables, particularly their experience of discrimination (OR = 1.46, *p* < 0.05).

As an additional analysis, we further explore whether the reasons for difficulties accessing health care services differ based on visible minority immigrant status. Overall, visible minority immigrants are more likely to report a range of reasons than non-visible minority native-born. For example, compared to non-visible minority immigrants (33%), visible minority immigrants (21%) are more likely to identify ‘getting a referral’ as difficult (OR = 1.22, *p* < 0.05). Similarly, visible minority immigrants (33%) are more likely to report ‘contacting physicians/nurses for information and advice’ as difficult than non-visible minority native-born (27%) (OR = 1.33, *p* < 0.001). Visible minority immigrants are also more likely to report ‘waiting between booking and visit’ as difficult than non-visible minority native-born (30%) (OR = 1.55, *p* < 0.001). In addition, compared to non-visible minority native-born (13%), visible minority immigrants (21%) are more likely to report ‘waited too long to get service’ as difficult (OR = 1.69, *p* < 0.001). Visible minority immigrants (8%) are more likely to report transportation problems than non-visible minority native-born (6%) (OR = 1.81, *p* < 0.001). Consistently, visible minority immigrants (3%) are also more likely to report language problems than non-visible minority native-born (1%) (OR = 2.68, *p* < 0.001). We also find that, compared to non-visible minority native-born (9%), visible minority native-born (12%; OR = 1.44, *p* < 0.05) and visible minority immigrants (11%; OR = 1.43, *p* < 0.001) are both more likely to report cost problems. Finally, non-visible minority (41%; OR = 79, *p* < 0.01) and visible minority immigrants (43%; OR = 0.85, *p* < 0.05) are less likely to report ‘not available at time required’ as difficult than non-visible minority native-born (46%).

## Discussion and conclusions

Difficulties accessing health care services can cause potential undesirable outcomes, such as delays in seeking and obtaining treatment and limited access to primary health care [3]. Although the Canada Health Act emphasizes universality and equity in the delivery of health care services, the research shows that difficulties accessing health care services are disproportionally burdened among vulnerable groups such as women, rural residents, Indigenous people, ACB people, and people with existing health problems [2, 3, 5, 6]. The current study advances the literature in two important ways. For one, guided by the theory of intersectionality, we extend our analysis to include the unique social locations linked to immigrant status and visible minority status in the context of difficulties accessing health care services. For another, it is equally important to extend our analysis to reflect the situation of the COVID-19 pandemic, considering that demand for health care system resources has been skewed by COVID-19 patients [1]. This situation may be increasing difficulties in accessing health care services among vulnerable populations such as visible minority immigrants. To this end, we compare difficulties accessing health care services among non-visible native-born, visible minority native-born, non-visible immigrants, and visible minority immigrants.

Depending on the types of health care services, we find that difficulties accessing health care services are commonly reported during the pandemic in Canada. Specifically, at least more than one-third of respondents face difficulties accessing dental care (48%), non-emergency surgical care (35%), non-emergency diagnostic test (39%), making an appointment with rehabilitative care (44%), medical specialist (46%), family doctor (37%), and seeking mental health care (41%). These figures are much higher than relevant statistics documented before the pandemic. For example, Harrington et al. [6] find that 22% of respondents in Ontario experience difficulties accessing specialist care for diagnosis and consultation. Similarly, research reveals that 16% report difficulties accessing non-emergency surgical care between 2005 and 2014 [5]. These findings potentially indicate that the COVID-19 pandemic is impacting Canadians’ access to specialist and routine care.

In addition, we find that compared to non-visible minority native-born, visible minority immigrants are less likely to report difficulties accessing non-emergency surgical care, non-emergency diagnostic test, dental care, mental health care, and making an appointment for rehabilitative care. These findings are very surprising because the literature indicates that access to health care among immigrants is most compromised in the use of preventive care, mental health care, and specialist care [5, 6, 20]. However, other scholars report comparable findings to our findings. For example, Setia et al. [15] show that female immigrants are less likely to report unmet care needs than their native-born counterparts. Similarly, Wu et al. [19] establish that the odds of reporting unmet care needs are lower for immigrants than the native-born, regardless of immigrants’ length of stay in Canada. It is also found that recent immigrants are less likely to report difficulties accessing preventive care services such as annual examinations [3]. There are at least two explanations for these findings. First, difficulties accessing health care services may be constructed based on people’s past experiences. It is possible that visible minority immigrants are perceiving fewer difficulties in Canadian health care settings, considering many of them come to Canada from less developed countries where there may be insurmountable barriers to accessing health care resources. In this context, visible minority immigrants may perceive that their access to health care services equals or surpasses that in their home countries. Second, as explained by Sanmartin and Ross [3], some visible minority immigrants may not have adequate levels of health literacy, which are necessary to identify the lack of appropriate health care. Specifically, some visible minority immigrants may still be in the process of learning to navigate the health care system sufficiently to get to the most suitable health care for themselves [21]. This potential bias may be underestimating their difficulties accessing health care services. As research shows that immigrants tend to underuse preventive health care services before the pandemic [22], it is possible that access to health care services has become even more complex with constant changes in health and safety protocols during the pandemic [23].

As opposed to these findings, we observe that visible minority immigrants are more likely to report difficulties accessing emergency services and urgent care since the beginning of the pandemic than non-visible minority native-born. Importantly, research shows that visible minority immigrants are more likely to lack a regular doctor than their white counterparts, regardless of their length of residence in Canada, although this difference is not observed among the native-born [24]. In addition, it is shown that recent immigrants who have been in Canada for fewer than 10 years are less likely to have family doctor than the native-born [25]. Similarly, Mian and Pong [26] observe that the chance of visiting emergency department is particularly high among recent immigrants in Canada. Although these findings indicate that emergency services and urgent care may be the only viable health care option for many visible minority immigrants, our results suggest that they seem to be exposed to unique barriers to access to these services since the beginning of the pandemic. For example, visible minority immigrants’ higher odds of reporting difficulties accessing emergency services and urgent care is partially explained by their experience of discrimination, implying that discrimination serves as a critical barrier to immediate care among visible minority immigrants. Considering that discrimination against visible minorities has been intensified during the pandemic [11], visible minority immigrants may be facing difficulties in accessing immediate care, leaving some of them with no option at all to address their health care needs.

We also observe the different patterns on the reasons for difficulties accessing health care services by visible minority immigrant status. For example, visible minority immigrants are more likely to report ‘getting a referral’ as difficult in comparison with non-visible minority native-born. Although access to speciality care often requires referrals from primary care providers in Canada, this process has been recognized as being complex among vulnerable populations [6]. Accordingly, getting a referral may be difficult among visible minority immigrants who often lack comprehensive knowledge about Canada’s health care system [16]. In addition, visible minority immigrants are more likely to report ‘waiting between booking and visit’ as their reason for difficulties accessing health care services. This result may imply that, wait time, which is often cited as one of the major structural barriers, is disproportionately affecting vulnerable populations such as visible minority immigrants during the pandemic. Visible minority immigrants are also more likely to report ‘contacting physicians/nurses to get information or advice’ and ‘language problems’ as barriers to health care services. These findings may be supported by research, arguing that health care services often lack culturally and linguistically competent care in Canada [27]. These barriers may be more intensified during the pandemic where in-person consultations have been largely replaced by virtual ones in many health care settings [28]. In addition, visible minority immigrants are more likely to report cost and transportation as problems than non-visible minority native-born. Despite the universal health care system, cost may still serve as a major barrier if they use uncovered health care services such as prescribing drugs and psychotherapy [6]. Similarly, as mentioned by Ahmed et al. [29], the cost associated with travel to and from health care facilities is often a concern among economically vulnerable groups including immigrants. Considering that visible minority immigrants are exposed to many unique reasons for difficulties, it may not be too surprising that they hesitate to use health care services in Canada, potentially leading to our observation that they are more likely to ‘wait too long to get service’ in comparison with non-visible minority native-born.

Based on these findings, we have several suggestions for policymakers with health equity in mind. Policymakers should move beyond a binary understanding of the social categorization of immigrant status (i.e., immigrants and the native-born), considering our findings that visible minority immigrants may be facing unique vulnerabilities and difficulties when it comes to accessing health care services during the COVID-19 pandemic. Specifically, multilevel actions are necessary to address the unique barriers that visible minority immigrants may be facing in accessing health care services in Canada. For example, at the structural level, it is important for policymakers to develop public policies that require organizations to collect disaggregated race-based data to inform programming. At the institutional level, evidence-based guidelines should be created to guide the implementation and promotion of culturally and linguistically competent care. These policy actions may reduce the level of barriers faced by racialized people and immigrants including language problems and racial discrimination experienced in health care settings. Moreover, community health education targeted visible minority immigrants may be helpful with opportunities to learn about Canada’s health care system comprehensively, which may enable them to learn about referral system and wait time. In addition, reflecting on the universal and equity principles embodied in the Health Canada Act, it is recommended that policy programs are implemented to reduce financial barriers derived from direct and indirect cost from health care access faced by structurally exposed groups including visible minority immigrants. These policy efforts possibly reduce difficulties accessing health care services among immigrants and visible minority immigrants, including our observation on visible minority immigrants’ difficulties accessing emergency services and urgent care.

Although our findings may be beneficial for policymakers, our study is not without limitations. For example, Statistics Canada’s Crowdsourcing Data series are useful for monitoring Canadians’ social and economic experiences during the pandemic. However, the data collection did not employ a probabilistic sampling technique. Therefore, our results are not generalizable to the Canadian population. Moreover, the data are cross-sectional in nature, limiting our results to statistical associations. Thus, caution is needed when interpreting the results. It is equally important to mention that access to health care services is socially desirable. In this sense, due to social desirability bias, difficulties in accessing health care services may be exposed to underreporting or overreporting. In addition, while we explore the intersectionality between immigrant status and visible minority status, it would have been useful to examine the heterogeneity of immigrants by length of residence in Canada (e.g., recent immigrants and established immigrants vs. native-born) and admission class (e.g., economic class, family class, and refugees). However, we are not able to employ this approach due to data limitations. Importantly, we are not able to control for any financial and economic status due to a lack of relevant information in the data, which could have been critical in further explaining the difference in difficulties accessing health care services based on visible minority immigrant status. We would also like to mention that there is a potential sampling bias that may have underrepresented visible minorities and immigrants in the sample. It is important to take into consideration that barriers to participation such as limited time and financial resources and language problems may be particularly relevant among vulnerable groups. This bias may have selected healthier visible minority immigrants who experience fewer unmet needs and barriers to many types of health care services. Finally, although we find that visible minority immigrants are less likely to report difficulties accessing a range of health care services, these results may be biased due to lack of adequate health literacy that helps evaluate the quality of health care services. As suggested by Etowa et al. [30], it is especially important to strengthen the collection and use of disaggregated data to understand the risk and burden of COVID-19 and to further unpack the underlying processes that contribute to the difficulties faced by immigrants and visible minorities in Canada in accessing health care services.

## Data Availability

Data are publicly available from https://search1.odesi.ca/#/.
